# Effect of suture technique on the occurrence of incisional hernia after elective midline abdominal wall closure: study protocol for a randomized controlled trial

**DOI:** 10.1186/s13063-015-0572-x

**Published:** 2015-02-15

**Authors:** René H Fortelny, Petra Baumann, Wolfgang E Thasler, Markus Albertsmeier, Stefan Riedl, Wolfgang Steurer, Jan Ludolf Kewer, Andreas Shamiyeh

**Affiliations:** Department of Surgery, Wilhelminenspital, Montleartstrasse 37, 1160 Vienna, Austria; Department of Medical Scientific Affairs, Aesculap AG, Am Aesculap Platz, 78532 Tuttlingen, Germany; Department of General, Visceral, Transplantation, Vascular and Thoracic Surgery, Grosshadern Hospital, Ludwig Maximilian University, Marchioninistrasse 15, 81377 Munich, Germany; Department of Surgery, ALP FILS Kliniken, Eichertstrasse 3, 73035 Göppingen, Germany; Department of Surgery, Robert Bosch Krankenhaus, Auerbachstrasse 110, 70376 Stuttgart, Germany; Department of Surgery, Klinikum Landkreis Tuttlingen, Zeppelinstrasse 21, 78532 Tuttlingen, Germany; Department of Surgery, AKh Linz, Krankenhausstrasse 9, 4021 Linz, Austria

**Keywords:** Abdominal wall closure, Incisional hernia, Laparotomy, Large bites, Small bites, Suture technique

## Abstract

**Background:**

Based on a recent meta-analysis, a continuous suture technique with a suture to wound length ratio of at least 4:1, using a slowly absorbable monofilament suture material, is recommended for primary median laparotomy closure. Incisional hernia, which develops in 9 to 20% of patients, remains the major complication of abdominal wall closure. Current clinical data indicate that the incidence of incisional hernias increases by 60% between the first and the third year after median laparotomy, implicating that a follow-up period of 1 year postoperatively is too short with regard to this common complication. Trauma to the abdominal wall can be reduced by improvements in suture technique as well as suture material. Several factors, such as stitch length, suture tension, elasticity, and tensile strength of the suture material are discussed and currently under investigation. A Swedish randomized controlled trial showed a significant reduction in the incisional hernia rate by shortening the stitch length. However, a non-elastic thread was used and follow-up ended after 12 months. Therefore, we designed a multicenter, international, double-blinded, randomized trial to analyze the influence of stitch length, using an elastic, extra-long term absorbable monofilament suture, on the long term clinical outcome of abdominal wall closure.

**Methods:**

In total, 468 patients undergoing an elective, median laparotomy will be randomly allocated to either the short stitch or the long stitch suture technique for abdominal wall closure in a 1:1 ratio. Centers located in Germany and Austria will participate. The primary endpoint measure is the incisional hernia rate 1 year postoperatively, as verified by ultrasound. The frequency of short term and long term complications as well as costs, length of hospital stay and patients’ quality of life (EQ-5D-5 L) will be considered as secondary parameters. Following hospital discharge, patients will be examined after 30 days and 1, 3, and 5 years after surgery.

**Discussion:**

This study will provide further evidence on whether a short stitch suture technique in combination with an elastic, extra-long term absorbable monofilament suture can prevent incisional hernias in the long term, compared with the long stitch suture technique.

**Trial registration:**

NCT01965249.

## Background

The risk of developing an incisional hernia after primary elective median laparotomy is reported in the literature as being between 9 and 20% [1]. Among multiple risk factors, the malfunction or delay of collagen synthesis appears to be of utmost relevance. Friedman *et al.* [2] showed direct correlation between a reduced collagen I/III quotient and the development of an unstable scar, based on immature, less mechanically stable, collagen 1. Obesity, steroid therapy, malnutrition, nicotine use, and connective tissue diseases are undeniably further main risk factors. But how should other factors, such as closure technique, suture material, or surgical experience, be interpreted in this context? Despite the paradigm shift in hernia surgery triggered in the beginning of the 1980s, which was initiated with the first documented use of plastic meshes by Usher [3], the problem of ensuring appropriate closure of the median laparotomy has not yet been satisfactorily solved. This issue still remains a topic of controversial discussion.

Since the publications of the Swedish group of Leif Israelsson [4,5] in 1993, the continuous closure technique with a defined suture/wound length ratio of 4:1 has increasingly been used [4,5]. For a long time, discussions regarding the ideal suture revolved around whether it should be ‘non- or slowly absorbable’. After numerous studies and meta-analyses [6-8], conducted most recently by Diener *et al.* [9], the evidence for the ideal primary abdominal closure seem to have been adequately established. Based on the most recent meta-analysis of primary laparotomy closure, the continuous suture technique with a suture to wound length ratio of at least 4:1, using a monofilament, slowly absorbable suture can be considered state of the art.

Minimization of trauma to the abdominal wall is the most important consideration in terms of improvement of surgical technique and suture material used. According to the biomechanical principles of abdominal wall tension, the distribution of suture tension over small tissue bridges using the appropriate needle size and suture strength to minimize tissue trauma is the most promising approach [10]. In addition to the surgical technique, the elasticity of the suture material is a further critical factor in preventing so-called ‘buttonholes’, according to physiological studies of the abdominal wall. Taking into account the results of experimental studies by Höer *et al.* [11,12], the association of different closure techniques and abdominal wall perfusions leading to different collagen ratios seems to be crucial in terms of creating a stable scar.

Based on these facts, the use of a monofilament suture material with a high elasticity in combination with a long-lasting basic retention strength as well as an extra-long absorption time of 390 days (MonoMax®, B. Braun Surgical SA, Rubi, Spain) seems to bear a significant potential for the improvement of abdominal wall suture stability [13]. This suture material might further improve the promising results of Millbourn’s randomized controlled trial [14] and will, therefore, by used in our multicenter, randomized controlled trial, which is aimed at comparing the long term clinical outcome of small versus large bite closure techniques after primary median laparotomies.

## Methods

### Study design

The Effect of Suture Technique on the Occurrence of Incisional Hernia (ESTOIH) study is a prospective, international, multicenter, double-blinded, randomized controlled trial, aimed at evaluating the efficacy of the short stitch suture technique using an elastic, extra-long term absorbable monofilament suture (MonoMax®) after primary median laparotomy, in comparison with the long stitch suture technique.

In total, 468 patients will be enrolled in seven centers located in Germany and Austria. Patients will be randomly allocated to receive either the short or the long stitch suture technique for abdominal wall closure in a 1:1 ratio. The surgical procedure used to close the abdomen will be standardized in both suture technique groups and the participating centers will be trained in the different suture techniques.

**Figure 1:**
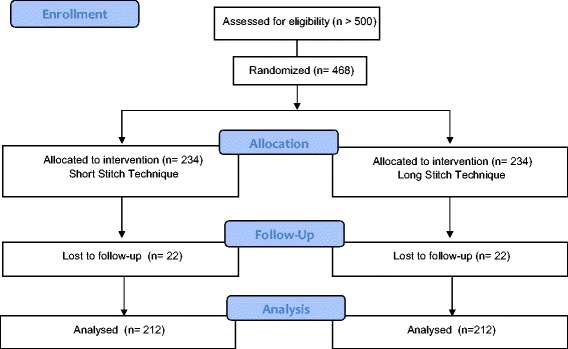
**CONSORT flow chart.**

**Figure 2:**
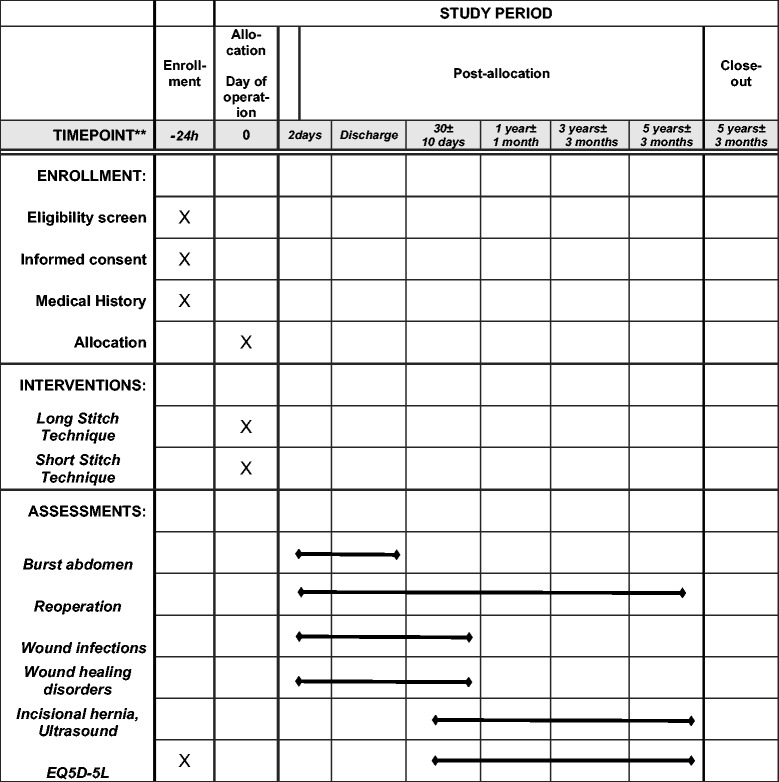
**SPIRIT diagram.** SPIRIT, Standard Protocol Items: Recommendations for Interventional Trials.

After the operative procedure, the investigator will examine the patients on the second day after surgery, on the day of discharge, and at 30 days ±10 days, 1 year ±1 month, 3 years ±3 months and 5 years ±3 months postoperatively. Patients and observers performing the follow-up visits will be unaware of the suture technique (double-blinded) used. The study will last 5 years for each patient (Figures 1 and 2).

### Participants

Patients at least 18 years of age undergoing an elective primary median laparotomy, with an incision length of ≥15 cm and an expected survival time longer than 1 year, and with an American Society of Anesthesiologists (ASA) status of I to III are eligible for this trial. After approval of the study protocol by the local ethics committee, patients will be screened consecutively for possible eligibility in the participating centers. All patients who seem to meet the in- and exclusion criteria will be asked whether they are willing to participate in the ESTOIH study and will be informed about the purpose of the trial. Written informed consent has to be obtained from each enrolled patient before the operation.

Exclusion criteria are:

- Emergency surgery,
- Body mass index ≥30 kg/m^2^,
- Pancreatic tumor patients,
- Patients who will be operated on owing to an abdominal aortic aneurysm,
- Peritonitis,
- Coagulopathy,
- Current immunosuppressive therapy (more than 40 mg of a corticoid per day or azathioprine),
- Chemotherapy within 2 weeks before operation,
- Radiotherapy of the abdomen within 6 weeks before operation,
- Pregnant women,
- Severe neurologic and psychiatric disease,
- Lack of compliance.

### Randomization

Eligible patients will be randomly allocated to receive either the short or the long stitch suture technique in a 1:1 ratio by opening a sealed opaque envelope. Envelopes are supplied by the sponsor, according to a randomization list provided by a statistician using the statistical software SAS 9.1. A separate randomization list was prepared for each participating center, to avoid center-specific effects and to assure a balanced distribution of both treatments within one center (stratification). Random blocks of different lengths were used. The randomization lists are sealed and locked up at the sponsor site. The envelopes will be assigned to the patients in chronological order by a surgeon, according to a consecutive random number.

Each envelope contains the device (suture material for the indented suture technique) as well as the information about which stitch technique should be applied for abdominal wall closure. Randomization is performed intraoperatively and briefly before abdominal wall closure. After randomization, each patient obtains a unique randomization number. The study site confirms the randomization result by sending a fax to the sponsor.

### Blinding

The treatment allocation is double-blinded. The patient and the observer responsible for the assessment of the clinical outcome will be unaware of the stitch length used for closing the midline and will have no access to the case report form. To document the clinical outcome of each follow-up examination, the sheets will be handed to the observer by an independent person (for example, a study nurse). The surgeons cannot be blinded because they have to know which suture length has to be applied for midline closure.

### Interventions

In both study groups, the abdominal wall closure will be performed using MonoMax® suture material (B. Braun Surgical SA, 08191 Rubi, Spain), an elastic, extra-long term absorbable, monofilament suture made of poly(4-hydroxybutyrate). In the long stitch group, a MonoMax® USP 1 loop in combination with a HR 48 needle will be used, and in the short stich group, a MonoMax® USP 2/0 single thread will be used in combination with a HR 26 needle. Details of the suture technique are shown in Table 1.

**Table 1:** **Comparison short stitch versus long stitch suture technique**

**Parameter**	**Long stitch technique**	**Short stitch technique**
**Suture material**		
Product	MonoMax®	MonoMax®
USP size	USP 1	USP 2/0
Length of thread	150 cm loop	150 cm single thread
Needle	HR 48	HR 26
**Suture technique**		
Stitch interval	10 mm	5 mm
Distance from median wound incision to stitch site	10 mm	5 to 8 mm
Suture length/wound length ratio	4:1	≥5:1
Suture technique	Continuous	Continuous

The surgical procedure is carried out as usual and according to local standards regarding the indication for intervention. During all operations, the umbilicus has to be completely dissected from the linea alba for an adequate abdominal wall closure. The linea alba is prepared free from fat and cut precisely in the middle. In the case of a deviation, this is recorded in the randomization fax. After abdominal wall closure, refixation of the umbilicus is achieved using an absorbable suture and in interrupted suture technique.

The following parameters are recorded and measured in both treatment groups:

- Length of the wound (cm),
- Number of threads used,
- Length of the remaining suture material (cm),
- Number of applied stitches,
- Duration for abdominal wall closure (defined as the time period from the first placement of the needle through the tissue until the last knot has been closed and the thread is cut).

Using these data, the suture length used to close the midline will be calculated, as well as the suture length to wound length ratio. The number of stitches will be analyzed in accordance with the wound length and the length of the implanted suture material. The distance between the stitches can be determined as well as the lateral distance from the incision.

### Implementation

Before the start of the trial, an initiation visit was paid to each center by the study management to inform and instruct the involved personnel on the study specific documents and procedures. To standardize both suture techniques, each participating center has been trained by presentations and videos; in addition, a practical workshop took place during the first study meeting. In three of the seven centers, surgeons had already used the short stitch technique before the start of the trial and have attended a hospitation conducted by the coordinating investigator in the Wilhelminenspital in Vienna, Austria. The other four centers were instructed during a visit by the coordinating investigator René H Fortelny before the first inclusions. All study centers were provided with suture material from the same batch, with the same type of stop watch for time measurement, a counter to enumerate the stitches, and rulers to measure the wound length, to standardize this equipment within the different centers.

### Outcome measures

#### Primary outcome measure

The primary endpoint of the study is the frequency of incisional hernias 1 year postoperatively, as assessed by ultrasound examination.

An incisional hernia is defined as an ‘abdominal wall gap with or without bulging in the area of a postoperative scar perceptible or palpable by clinical examination or imaging’ according to the European Hernia Society. Incisional hernias will be classified according to their localization and size.

#### Secondary outcome measures

The length of hospital stay, cost, and quality of life, and the frequency of short term and long term complications will be compared between the short stitch and the long stitch group.

The length of hospital stay is defined as the time period from the day of surgery until the day of discharge.

The assessment of costs includes the material cost (suture), cost per operation minute (duration for abdominal wall closure), cost of hospital stay, and the cost saving per prevented incisional hernia.

To analyze quality of life, the EQ-5D-5 L questionnaire will be used and documented by the patient preoperatively, and after 30 days, 1 year, 3 years, and 5 years postoperatively. The EQ-5D-5 L is used in the German language version and a license has been obtained from the EuroQol Group by the sponsor. The EQ-5D is a standardized measure of health status developed by the EuroQol Group to provide a simple, generic measure of health for clinical and economic appraisal. The questionnaire is designed for self-completion by respondents and takes only a few minutes to complete. Instructions to respondents are included in the questionnaire. The EQ-5D-5 L consists of two pages a descriptive system and the EQ Visual Analogue Scale (EQ-VAS). The descriptive system comprises five dimensions (mobility, self-care, usual activities, pain or discomfort, anxiety or depression). Each dimension has five levels: no problem, slight problems, moderate problems, severe problems, and extreme problems. The EQ-VAS records the respondent’s self-rated health on a 20 cm vertical visual analogue scale with endpoints labeled ‘the best health you can imagine’ and ‘the worst health you can imagine’.

Short term complications include the frequency of burst abdomen and the reoperation rate due to burst abdomen until day of discharge. Wound infections occurring until the day of discharge and within 30 days after surgery will be recorded as short term complications. Wound infections are defined according to the US Centers for Disease Control and Prevention (CDC). Furthermore, wound healing complications, such as seroma, necrosis, and fistula, reported until 30 days postoperatively will be counted as short term complications.

Long term complications include the frequency of incisional hernias after 3 and 5 years after surgery, ad detected by ultrasound examination. Furthermore, wound infections according to CDC classification 1 year postoperatively and wound healing complications occurring within 1 year after surgery will be investigated.

### Sample size calculation

The ISSAAC study has shown a risk of 19% of developing an incisional hernia within one year using MonoMax® suture material and a long stitch technique for abdominal wall closure of a primary elective midline laparotomy [14]. The aim of this study is to demonstrate that the short stitch suture technique with MonoMax® will decrease the incisional hernia rate after 1 year by 50% compared with the long stitch technique (primary endpoint). Considering hernia rates of 19% and 9.5% in both groups, a total sample size of 424 patients (212 per group) will be necessary to detect this difference with a power of 80% and an alpha of 5%. Including a drop-out rate of 10%, a total of 468 patients will be randomized in the current study. Thus, each of the seven participating centers should recruit about 67 patients. To avoid center effects, a maximum of 200 patients may be recruited per center. Withdrawn patients will not be replaced.

### Statistical analysis

A planned analysis will be performed after all patients have completed their 1 year follow-up (primary endpoint). All data available until 1 year follow-up will be analyzed. Additional analyses will be conducted after completion of the 3 and 5 year follow-ups. It is intended to publish the 1 year result as well as the 3 and 5 year results in a peer-reviewed journal.

The statistical analysis of the primary endpoint has a confirmative character, whereas the secondary endpoints will be analyzed exploratively.

The description of the patient cohort with respect to demographic data and the baseline values of investigated parameters will take place as a whole as well as grouped by treatment group.

### Primary endpoint

The study hypothesis will be tested by a two-sided chi square test for independent proportions.

The hernia rate is calculated as a Kaplan-Meier estimate for the failure rate at the end of the observation period (1 year ± 1 month). The Kaplan-Meier estimate has the benefit of additionally taking into account the results of drop-out patients. For each hernia, the date of its first observation must be documented to provide a correct estimate. The date of hernia detected by ultrasound at a follow-up examination will be set to the examination date; this may lead to a slight over-estimation of the hernia rates in both groups.

Analyses will be performed according to the intention-to-treat principle. The consistency of the results will be tested by per-protocol analysis (sensitivity analysis).

### Secondary endpoints

Secondary endpoints will be tabulated as frequencies and rates, or as means and standard deviations, as appropriate. Confidence intervals will be used when appropriate.

The two-sided chi square test for independent proportions will be used for proportion comparisons. The Kaplan-Meier method will also be used to calculate other rates to be analyzed. For each such event, the date of occurrence must be recorded. The two-sided *t* test for independent samples will be used for comparisons with continuous variables.

### Safety

Descriptive statistics methods will be used to analyze safety (listings and, when appropriate, frequency tables of relevant events by treatment group).

### Ethical and legal issues

The final study protocol was approved by the ethics committee of Vienna, Austria. Secondary approval was obtained from all local ethics committees responsible for the participating centers:

- Ethics committee of Vienna, Austria,
- Ethics committee ‘Landes Oberösterreich’, Linz, Austria,
- Ethics committee ‘Bundesland Salzburg’, Salzburg, Austria,
- Ethics committee of Ludwig Maximilian University, Munich, Germany,
- Ethics committee ‘Landesärztekammer Baden Württemberg’, Stuttgart, Germany,
- Ethics committee of University Tübingen, Germany.

Any substantial changes to the original documents will be submitted to the ethics committees in line with pertinent regulatory requirements.

This study will be carried out in accordance with the principles of the Declaration of Helsinki and in compliance with Good Clinical Practice. The trial staff will ensure the preservation of the pseudonymity of the patients. Upon inclusion in the study, each patient will receive a unique consecutive patient randomization number. The number consists of a five-digit code. The first two digits indicate the center and the following three digits stand for the sequential patient number.

### Quality control and assurance

Quality control will involve collecting data on adherence to the intervention, patient inclusion and follow-up, as well as monitoring the quality of data entry. Authorized qualified representatives of the sponsor will visit the participating clinics at regular intervals, as defined in the monitoring plan, to verify adherence to the protocol and local legal requirements, to perform source data verification, and to assist the investigator in study-related activities. Up to ten monitoring visits per center are planned, depending on the number of recruited patients.

Data will be entered in a database, as recorded in the paper-based case report form. To ensure data quality, double entry will be used. After completion of data entry, checks for plausibility, consistency, and completeness will be performed. Based on these checks, queries will be produced combined with queries generated by visual control. All missing data or inconsistencies will be reported to the centers and clarified by the responsible investigator.

## Discussion

For several generations of surgeons, the mass-layer single stitch suture has been the most common and standard technique for closure of the median incision. There is limited evidence that even with the ‘long stitch’ technique using a loop suture, only one layer, the fascia only, should be included [15] because there is less trauma to the tissue, implying a reduction in the risk of a burst abdomen. In addition to mesh development, today, different suture materials are available for closing the abdominal wall in primary laparotomies and incisional hernia surgery. Experimental studies and the clinical adaptation of an innovative ‘short stitch’ technique by the Israelsson study group [14,15] clearly proved, in a controlled trial, the concept of reduced tissue trauma and the impact on infection and incisional hernia rates when using this technique. Reducing the distance between the stitches and the wound edge to between 5 and 8 millimeters, thereby increasing the suture/wound length ratio, and using slowly absorbable sutures of size USP 2/0 (polydioxanone) and a half-circle round-bodied needle (thread length: 20 mm; arch length: 31 mm) and grasping only the aponeurosis, led to a significant decrease in the infection rate, as well as in the incisional hernia rate, compared with the ‘long stitch’ group. The average suture/wound length ratio of the ‘short stitch’ group of 5.7 versus 6.4 percent in the ‘long stitch group’ resulted in a significantly lower rate of wound infection of 5.2 versus 10.2 percent, as well as a highly significant lower incisional hernia rate of 5.6 versus 18 percent at the one-year follow-up. The multivariate analysis revealed a twofold risk of wound infection and a fourfold risk of incisional hernia occurring in the ‘long stitch’ group. Another randomized controlled trial, the STITCH study [16] obtained similar significant results when comparing the large versus short stitch technique of primary midline closure. The one-year results of this study were presented at the annual EHS-Meeting in Edinburgh in May 2014. The incisional hernia rate in the large stitch group was 23%, compared with 14% for the short stitch group. In comparison with the Millbourn study [14], the incisional hernia rate is more than twice as high in the short stitch group, using an identical suture material. The reason for this difference might be the complete follow-up of all patients by ultrasound. An increase in the incisional hernia rate by 60% from 12 months to 36 months postoperatively has to be taken into account, as detected in the recently published data by Fink *et al.* [17], regarding the long term follow-up of the INSECT and ISSAAC trials [18,13].

The significant advantage of the short stitch technique in causing minimal trauma to the abdominal wall seems to be clearly supported by the evidence-based level, whereas the synergetic effect of a special suture material used in this field has yet to be proven.

Based on the use of an elastic suture material with a long-lasting strength retention and ultra-long absorption time (MonoMax®), the ESTOIH study can be expected to yield lower hernia rates than the Millbourn study [13].

### Trial status

The study protocol was been registered on 9 October 2013. A first investigator meeting was held on 28 February 2014 in Munich, Germany. All centers have been initiated and are actively recruiting. The first patient was recruited on 6 March 2014. In total, 55 patients were randomized, while the manuscript was being completed. Recruitment is expected to end in December 2015.
